# Dysfunctional Hyperpolarization-Activated Cyclic Nucleotide-gated Ion Channels in Cardiac Diseases

**DOI:** 10.5935/1678-9741.20160030

**Published:** 2016

**Authors:** Xiaoqi Zhao, Tianxiang Gu

**Affiliations:** Department of Cardiac Surgery ICU, The First Affiliated Hospital of China Medical University, Shenyang, P.R. China

**Keywords:** Ion Channel Gating, Cardiac Electrophysiology, Electrophysiological Processes.

## Abstract

Hyperpolarization-activated cyclic nucleotide-gated (HCN) channels are reverse voltage-dependent, and their activation depends on the hyperpolarization of the membrane and may be directly or indirectly regulated by the cyclic adenosine monophosphate (cAMP) or other signal-transduction cascades. The distribution, quantity and activation states of HCN channels differ in tissues throughout the body. Evidence exhibits that HCN channels play critical roles in the generation and conduction of the electrical impulse and the physiopathological process of some cardiac diseases. They may constitute promising drug targets in the treatment of these cardiac diseases. Pharmacological treatment targeting HCN channels is of benefit to these cardiac conditions.

## INTRODUCTION

The hyperpolarization-activated cyclic nucleotide-gated (HCN) channels comprise a family of cation channels activated by hyperpolarized membrane potentials and stimulated by intracellular cyclic nucleotides. The members of the family are four human isoforms (HCN1-4). Upon hyperpolarization, all four isoforms generate a inward current (If) in the heart and in the nervous system^[1-3]^. The hyperpolarization-activated funny current (If) is a major determinant of cardiac diastolic depolarization and plays a key role in controlling heart rate^[4]^. The expression of HCN channel is age-related, the expression has difference in distinct growth stages, HCN genes are inactivated in ventricular myocytes cells following maturation whereas they expressed in these cells in the fetal and/or neonatal heart^[5-8]^. Three isoforms (HCN1, HCN2, HCN4) are expressed in cardiac tissues, with HCN2 and HCN4 the dominant subtypes. In the healthy adult heart, HCN channels are predominantly expressed in the conduction system, especially in the sinoatrial node, HCN4 has been determined as the principal HCN isoform in sinoatrial node cells. However, some studies demonstrate a significant expression of HCN1 and also a small amount of HCN2 beneath HCN4 in murine sinoatrial cells at the protein level, indicating that the three subunits together contribute to If in these cells^[8,9]^. HCN2 has long been known to be expressed in cardiac ventricular myocytes^[10]^, and recently, the expression of HCN4 and low amounts of HCN1 and in addition a functional role of HCN3 in ventricular myocytes has been demonstrated^[11,12]^, but the expression of HCN channels in healthy adult ventricular myocardium is generally weaker than in the conduction system, so If currents are rarely detectable in normal ventricular myocytes^[13,14]^. The kinetics of HCN1 is the fastest, followed by HCN2, and the slowest is HCN4^[15]^. cAMP can make the potential of HCN channel open probability curve more positive. HCN2 and HCN4 channels increased 10-20 mV, and HCN1 only 5 mV. HCN channel is also affected by the endocrine system. Studies have demonstrated that HCN2 and HCN4 gene transcription is controlled by thyroid hormone, so it is speculated that HCN2 and HCN4 gene down-regulation level is an important mechanism for bradycardia induced by hypothyroidism. Positive inotropic effect of thyroid hormone may be associated with upregulation of the channel molecules^[16,17]^.

## HCN AND CARDIOMYOPATHY

A large number of studies have found that in heart failure, atrial fibrillation, myocardial hypertrophy and myocardial infarction, atrial and ventricular HCN2 and HCN4 channels gene express unusually causing atrial or ventricular myocyte If current rise or fall, and all of these may be associated with heart disease fatal arrhythmia^[18]^. Under normal conditions, HCN channels are poorly expressed outside the cardiac pacemaking and conduction system^[11,18-20]^, but it changes during cardiac disease. During hypertension and heart failure, If activity is observed in the ventricular myocardium due to re-expression of HCN genes^[18,21-23]^, several studies proved that If current density and occurrence is significantly greater in hypertrophic cardiomyocytes and end-stage failing hearts and this is directly related to the arrhythmias^[18,21,24-27]^. Basing on the presence of If current in ventricular myocytes isolated from severely hypertrophied rat hearts, the current arrhythmogenic role in cardiac hypertrophy and failure has been inferred^[25]^, and its density is larger in human ventricular myocytes isolated from the hearts of patients with ischemic than in those with dilated cardiomyopathy^[21]^. Stillitamo et al.^[18]^ demonstrated for the first time comparing the mRNA and protein expression of HCN subunits in the human atrial and ventricle under normal and heart failure conditions in human heart failure, an upregulation of ventricular HCN2 and HCN4 underlies the increase in functional If current, Michael et al.^[28]^studied the model with ventricular HCN2/4 knockout and identified the enhanced If as an important contributor to the typical electrophysiological alterations observed in the hypertrophic heart, including prolonged ventricular action potentials and lengthened QT intervals. So the overexpression of If may contribute to increased ventricular (and atrial) propensity to arrhythmias which leading to sudden death caused by ventricular tachycardia or fibrillation in heart failure and hypertrophic heart. Cardiac dysfunction is a complex and important problem for people with epilepsy. Recently, a study has showed that cardiac electrophysiology was significantly altered in adult rat models of genetic or acquired epilepsy, with slower heart rate, shorter QRS duration, longer QTc interval, greater standard deviation of RR intervals, and bradycardia, molecular analysis demonstrated significant reductions in cardiac HCN2 mRNA and protein expression in models, providing a molecular correlate of these electrophysiologic abnormalities^[29]^.

## HCN AND ARRHYTHMIAS

HCN channels are essential for cardiac pacemaker and electric conduction. The cardiac hyperpolarization activated cation current (If) is known to be present in region with primary or second pacemaker activity, which is found in nonpacemaking regions of the heart. In pacemaker regions, If is believed to contributed to spontaneous diastolic depolarization. HCN1-knockout animals display a dysfunction of the sinoatrial node resulting in sinus dysrhythmia, sinus pauses and a reduced cardiac output, demonstrating that HCN1 contributes to a stable cardiac rhythm generation^[30]^. Atrial fibrillation is the most common sustained tachyarrhythmia and is one of the major causes of cerebrovascular accident^[31,32]^. Clinical electrophysiology studies in patients have demonstrated that rapid focal activity originating from PV can trigger and maintain atrial fibrillation^[33-35]^. A study found that the pacing-induced spontaneous cation potential and larger If current were observed in the PVs cardiac myocytes from canine using RAP (rapid atrial pacing), and a slow diastolic depolarization in the PVs cells. Meanwhile If current densities were significantly higher. As mentioned above, delayed ventricular repolarization and prolonged QT intervals exist in hypertrophic heart, and they can promote early afterdepolarizations and electrical instability, so ventricular HCN channels may play a significant role in the diseased heart by increasing the risk for severe ventricular arrhythmias^[36,37]^. For the patients with advanced atrioventricular block or sick sinus syndrome, pacemakers are implanted. Biological pacemakers have several advantages (improved cardiac output, no need for periodic resizing in a child during maturation, among others), so the efforts to use gene therapy to create a biological pacemaker have been ongoing for many years. And most of the efforts focused on HCN gene family of channels. Plotnikov et al.^[38]^ has proved that HCN212 chimeric channel is noteworthy, therefore it resulted in tachycardia when overexpressed in the canine left bundle branch to achieve the desired physiological range.

## HCN CHANNEL BLOCKERS

It is well known that slow heart beat is good for the heart. Therefore, HCN channels become the main target of the drug study controlling rapid arrhythmia. Recently, there are many studies about ivabradine, the first selective blocker of If current. A work done by Kuwabara et al.^[39]^ analyzed the effect of ivabradine on survival and rhythmicity in a transgenic mouse model (dnNRSF) of dilated cardiomyophathy, and the founding is although an increased cardiac expression of If accompanied by ventricular tachyarrhythmias and sudden arrhythmic death, the survival was improved significantly when ivabradine was administrated at low doses without affecting heart rate. Ivabradine can markedly reduce If currents in the RAP (rapid atrial pacing) cells in a concentration-dependence manner^[40]^. It has been proved that Ivabradine can reduce heart rate without exerting negative inotropic or vasodilatory effects^[41]^. A recent clinical trial, the Systolic Heart failure treatment with the If inhibitor ivabradine Trial (SHIFT), showed the outcomes of patients with resting heart rates ≥70 bpm were significantly improved with the heart rate decreased^[42]^. A subgroup analysis of BEAUTIFUL trial (morbidity-mortality evaluation of the If inhibitor ivabradine in patients with coronary artery disease and left ventricular dysfunction study) suggested a specific benefit for patients with heart rates over 70 bpm^[43]^, and echocardiography revealed that the reduction in the left ventricular systolic volume index observed in the ivabradine treatment was related to the degree of heart rate reduction, although the large study showed the composite endpoint was not significantly different between the ivabradine and the placebo groups. All of the results suggest that ivabradine improves cardiac diseases outcome depends on its reduction of heart rates. In the treatment of chronic heart failure, β-receptor blockers are used widely, but some patients cannot tolerant β-receptor blockers (because β-blockers may induce asthma, negative inotropic effect and so on), thus, ivabradine maybe the alternative. Another study examined the effects of ivabradine on survival and arrhythmicity in transgenic mice (dnNRSF-Tg). In this study, ivabradine was used 7 mg/kg per day orally, and the results showed it significantly reduced ventricular tachyarrhythmias and improved survival among dnNRSF-Tg mice while having no significant effect on heart rate or cardiac structure or function. Ivabradine inhibited the abnormal automaticity induced by β-adrenergic stimulation and prevented the increase in automaticity in dnNRSF-Tg mice^[39]^. It is well known that the incidence of sinoatrial node dysfunction increases with age and is accompanied by atrial fibrillation (AF) and other tachyarrhythmia^[44]^. AF is one of the most common arrhythmias. It is estimated that 84% of the patients with AF are over 65 years old, and the incidence rate of AF doubles every 10 years, the incidence rate of AF is 10% in people over 85 years old. It can lead to congestive heart failure, thromboembolism, and cerebrovascular accident. So the mortality rate of AF is very high^[45,46]^. Recent animal study showed that dogs with agerelated AF induced by rapid atrial pacing administered ivabradine for intervention, and ivabradine could increase the effective refractory periods in the left atrium and left superior pulmonary vein, reduce the duration of AF after induction, and significantly decrease the inducing rate of AF (the incidence rate of AF decreased from 50% to 25%). Therefore, ivabradine has potential effects in preventing age-related AF^[47]^. Amiodarone is one of the most frequently used anti-arrhythmic drugs. Amiodarone was shown to inhibit HCN channel currents in oocytes and HEK-cells. To determine the effects of amiodarone under pathological conditions, a study monitored If under the amiodarone treatment in ventricular myocytes from spontaneously hypertensive rats with left ventricular hypertrophy using the whole-cell patch-clamp technique. The findings of the study showed that the incubation of myocytes with amiodarone significantly suppressed If density and downregulated HCN2 and HCN4 expression at both the mRNA and protein level. This is the first study indicating that amiodarone inhibits If under hypertrophied condition^[48]^.

## PERSPECTIVES

With the development of the study on HCN channels, the importance of HCN channels on heart diseases attracted more attention. The biological pacemaker based on HCN channels has attached much attention, and a lot of *in vitro* and *in vivo* gene transduction experiments proved the feasibility of constructing cardiac biological pacemaker, but its clinical application and safety issues is yet to be confirmed further. In order to avoid side effects of HCN channel blockers, the subtype-selective HCN channel blocker is promising.
